# Borderline personality disorder and risk of atrial fibrillation: insights from a bidirectional Mendelian randomization study

**DOI:** 10.3389/fpsyt.2024.1392605

**Published:** 2024-07-10

**Authors:** Wenzhe Zhou, Zhimiao Wang, Hesheng Hu, Yugen Shi, Qiubo Wang, Mei Xue

**Affiliations:** ^1^ Shandong Provincial Maternal and Child Health Care Hospital, Jinan, China; ^2^ Department of Gerontology, The First Affiliated Hospital of Shandong First Medical University & Shandong Provincial Qianfoshan Hospital, Jinan, China; ^3^ Department of Cardiology, The First Affiliated Hospital of Shandong First Medical University & Shandong Provincial Qianfoshan Hospital, Jinan, China; ^4^ Shandong First Medical University & Shandong Academy of Medical Sciences, Jinan, China

**Keywords:** borderline personality disorder, atrial fibrillation, casual association, bidirectional, Mendelian randomization

## Abstract

**Background:**

Atrial fibrillation (AF) is one of the most common form of arrhythmia. Previous studies have shown a link between AF and mental illness. However, the causal relationship between mental illness and AF remains unclear. The purpose of this study was to investigate the bidirectional causal relationship between borderline personality disorder (BPD) and AF.

**Method:**

We used the bidirectional Two-sample Mendelian randomization (TSMR) method to evaluate the causal relationship between BPD and AF. Instrumental variables associated with BPD were derived from a genome-wide association study involving 214,816 Europeans (2,637 cases and 212,179 controls). We then obtained atrial fibrillation data from the GWAS meta-analysis (60,620 cases and 970,216 controls). The TSMR analyses were performed in five methods, namely fixed-effect inverse-variance weighted (IVW) method、random-effect IVW method, MR Egger regression method, Weighted median method and Simple mode method. Several sensitivity analyses are used to test the robustness of positive results.

**Results:**

The fixed-effect inverse-variance weighted model [Odds ratio (OR), 1.033, 95% confidence interval (CI), 1.011-1.056, *P* = 0.0031], random-effect inverse-variance weighted model (OR, 1.033; 95%CI, 1.005-1.062; *P* = 0.0191) and Weighted median (OR, 1.034; 95%CI, 1.002-1.068; *P* = 0.0394) all showed that genetically predicted BPD was associated with an increased risk of AF. Sensitivity analysis using other MR Methods, including the MR-Egger intercept, MR-Presso method, and leave-one-out analyses, showed that the results were robust. In reverse MR analysis, there was no causal relationship of AF on BPD.

**Conclusion:**

Our study provides a causal relationship between BPD and AF. This means that patients with BPD should be monitored for the occurrence of AF. Early screening and proper management of BPD may show anti-arrhythmic benefits.

## 1 Introduction

Atrial fibrillation (AF) is one of the most common arrhythmias in clinic. It is characterized by rapid and disordered atrial electrical activity. AF can be divided into first diagnosed AF, paroxysmal AF, persistent AF, and permanent AF (1). AF can make people experience a variety of uncomfortable symptoms such as panic, fatigue, sweating, which seriously affect their quality of life (2). The purpose of AF treatment is to prevent thromboembolic complications, restore and maintain sinus rhythm, and control ventricular rate during AF (3). The most important thing in the treatment of AF is to actively search for the primary disease and inducing factors, and make corresponding treatment. Studies have shown that male sex, advancing age, and Caucasian ancestry are important risk factors for developing AF (4). Controllable risk factors for AF include smoking (5), alcohol use (6), high blood pressure (7), diabetes (8), obesity (9), obstructive sleep apnea (10), and a sedentary lifestyle (11), among other factors. Previous studies have shown that AF and psychiatric disorders are inextricably linked. It is important to note that patients with AF often exhibit one or more negative emotions, such as anxiety, depression, reactive instability, and high neuroticism under the influence of severe stressors (12). It has been demonstrated that depression significantly increases the cumulative incidence of atrial fibrillation (from 1.92% to 4.44% at 10 years), and 20-40% of AF patients are found to have high levels of depression (13). Similarly, Kim et al. found that depression was associated with a significantly increased risk and cumulative incidence of new-onset AF (14). A meta-analysis of cohort studies demonstrated that anxiety was independently associated with an increased risk of recurrence of AF after catheter ablation (adjusted relative risk, 2.3; 95%CI, 1.710-3.260; *P* < 0.001) (15). One study demonstrated that the level of anxiety before coronary artery bypass surgery is an important factor in the development of AF after surgery (16). Studies have shown that severe psychological distress (35%) and suicidal ideation (20%) were prevalent in the tertiary population with AF (17).

Mental illness causes serious social and economic burden, and it is difficult to accurately identify its pathogenesis and risk factors (18). Due to the complex pathogenesis of mental diseases, the reverse causality between risk factors and mental diseases is easily confused, which makes MR analysis become an important tool in the study of mental diseases. A previous review of 50 MR articles showed a causal relationship between a particular psychiatric disorder and its causative factors (19). This review is divided into four groups based on the mental disorders assessed: schizophrenia, major depressive disorder, attention deficit and hyperactivity disorder, and autism spectrum disorder or other mental disorders. BPD, though one of the screening criteria in this review, was not included in the target article. It can also be seen that the number of MR articles on BPD is relatively small. To some extent, our paper can fill the gap in the study of MR of BPD. Borderline personality disorder (BPD), also known as emotionally unstable personality disorder, is characterized by widespread instability in interpersonal relationships, emotional regulation, impulse control, and self-image management (20). Patients with BPD often suffer from depression, anxiety, impulsivity, anger and other negative emotions, and even have self-harm and suicidal tendencies (21). Emotional instability is the main characteristic of BPD. BPD is a serious mental disorder that can have a significant impact on an individual’s quality of life, mental health and social interactions. We hypothesize that there may be a causal relationship between BPD and AF.

Mendelian Randomization (MR) is a data analysis technique used to evaluate causal inference in epidemiological studies. In MR analysis, genetic variants are used as Instrumental Variables (IVs) to estimate the causal relationship between the exposure factor of interest and the outcome of concern (22). We conducted a two-sample bidirectional MR study to estimate the causal relationship between BPD and AF. This paper expounds the causal relationship between BPD and AF, and provides the basis for early prevention and detection of arrhythmia in patients with BPD.

## 2 Method

### 2.1 Study design and data sources

In this study, bidirectional two-sample Mendelian randomization (TSMR) was used to evaluate the causal relationship between BPD and AF.

The summary data for BPD were derived from a recently published genome-wide association study (GWAS) that included 2,637 patients with BPD and 212,179 control patients (https://gwas.mrcieu.ac.uk/datasets/finn-b-F5_EMOPER/). We used the most recent GWAS meta-analysis of AF by Nielsen et al., which included 60,620 patients with AF and 970,216 controls of European ancestry (https://gwas.mrcieu.ac.uk/datasets/ebi-a-GCST006414/). The analysis pooled six contributing studies [The Michigan Genomics Initiative (MGI), deCODE, the Nord-Trøndelag Health Study (HUNT), DiscovEHR, UK Biobank, and the AFGen Consortium] (23). Detailed information about the data sources is shown in **Table 1**.

**Table 1:** Detailed information of studies and datasets used for analyses.

	Exposure (outcome): BPD	Outcome (exposure): AF
Year	2021	2018
Data source	FinnGen	Nielsen et al.
Ethnicity	European	European
Sample size	214,816	1,030,836
cases	2,637	60,620
controls	212,179	970,216
Number of SNPs	16,380,456	33,519,037
GWAS ID	finn-b-F5_EMOPER	ebi-a-GCST006414
Web source	https://gwas.mrcieu.ac.uk/datasets/finn-b-F5_EMOPER/	https://gwas.mrcieu.ac.uk/datasets/ebi-a-GCST006414/

BPD, borderline personality disorder; AF, atrial fibrillation.

### 2.2 Three core assumptions

**Figure 1:**
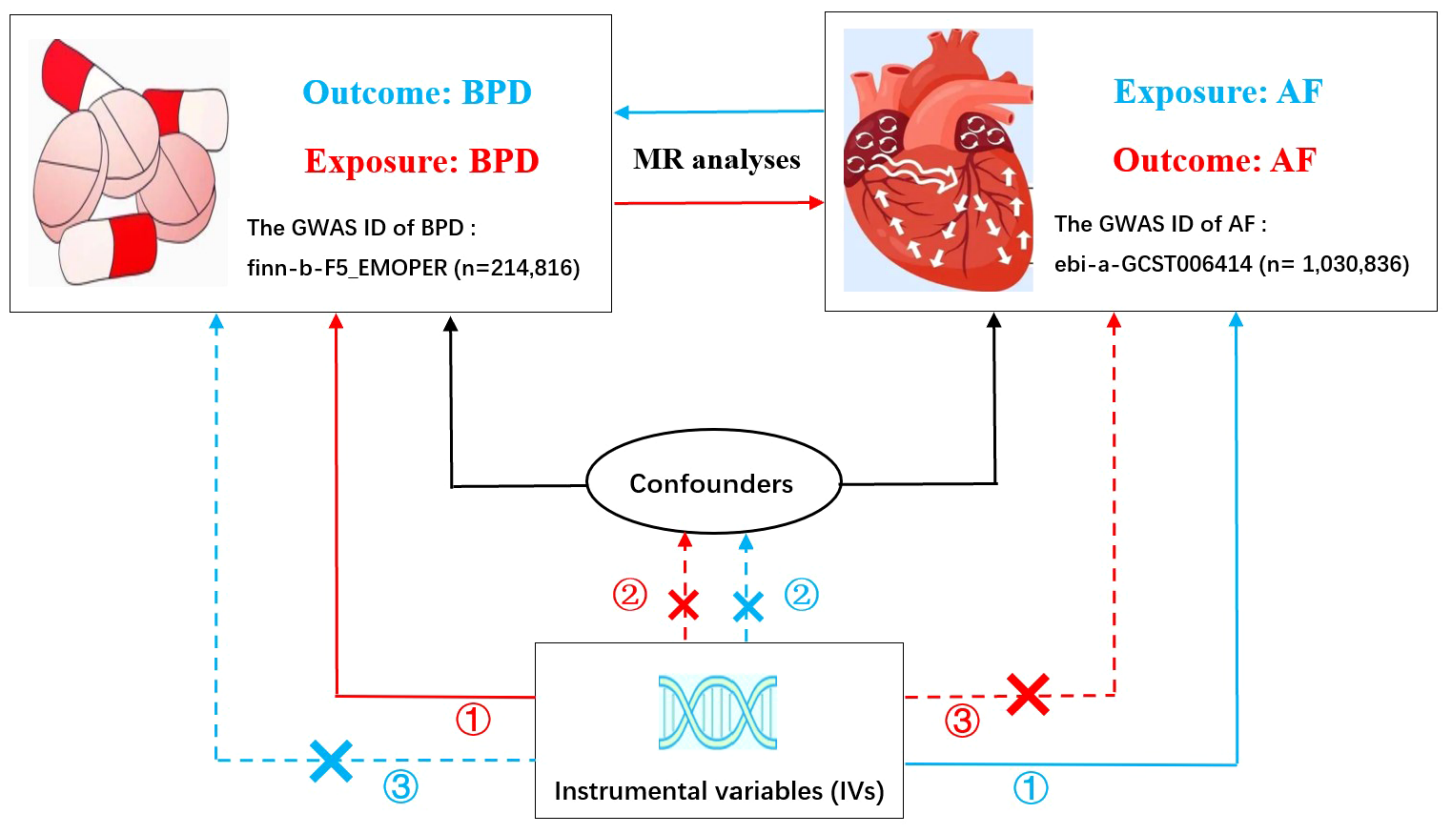
Bidirectional Mendelian randomization model of BPD and AF. (The red line represents BPD as exposure and AF as outcome; The blue line represents AF as exposure and BPD as outcome.) BPD, borderline personality disorder; AF, atrial fibrillation; GWAS, genome-wide association study. Three crucial hypotheses of the Mendelian randomization study: ① the correlation assumption; ② the independence assumption; ③ the exclusion assumption.

In MR analysis, three core assumptions must be satisfied in order to obtain valid results (24), as shown in **Figure 1**. Specifically, multiple genetic variants must satisfy: (1) the correlation assumption: IVs are closely related to exposure; (2) the independence assumption: IVs are not associated with other confounding factors; (3) the exclusion assumption: IVs only affect the outcome through the exposure.

### 2.3 Selection and validation of SNPs

In this study, in order to obtain more screening data, we used *P* < 5×10^-6^ as the criterion for screening SNPs for BPD at the genome-wide level (25). In the genome range, 16 gene loci were identified that were significantly associated with BPD. Similarly, we found 111 independent genetic loci for AF that achieved genome-wide significance (*P* < 5×10^-8^). Linkage disequilibrium (LD) refers to a nonrandom association between allele of different loci, measured using two parameters, r^2^ and kb. The LD window is set to 10000kb, r^2^ > 0.001 to ensure the independence of the selected genetic variation. F-statistic of genetic variation 10, the genetic tool is considered to be effective, that is, it is not affected by weak instrument bias (26). The calculation formula is: F=R²*(N-2)/(1-R²). R²=2*(1-MAF)*MAF*β². R² is the degree of variation explained by each SNP, EAF is the minor allele frequency, β is the beta coefficient associated with exposure, and N is the total sample size. Prior to MR analysis, we performed effective allelic comparisons to remove all SNPs with palindromic structures. This gives us a standard instrumental variable that conforms to MR analysis.

### 2.4 MR analysis and sensitivity analysis

**Figure 2:**
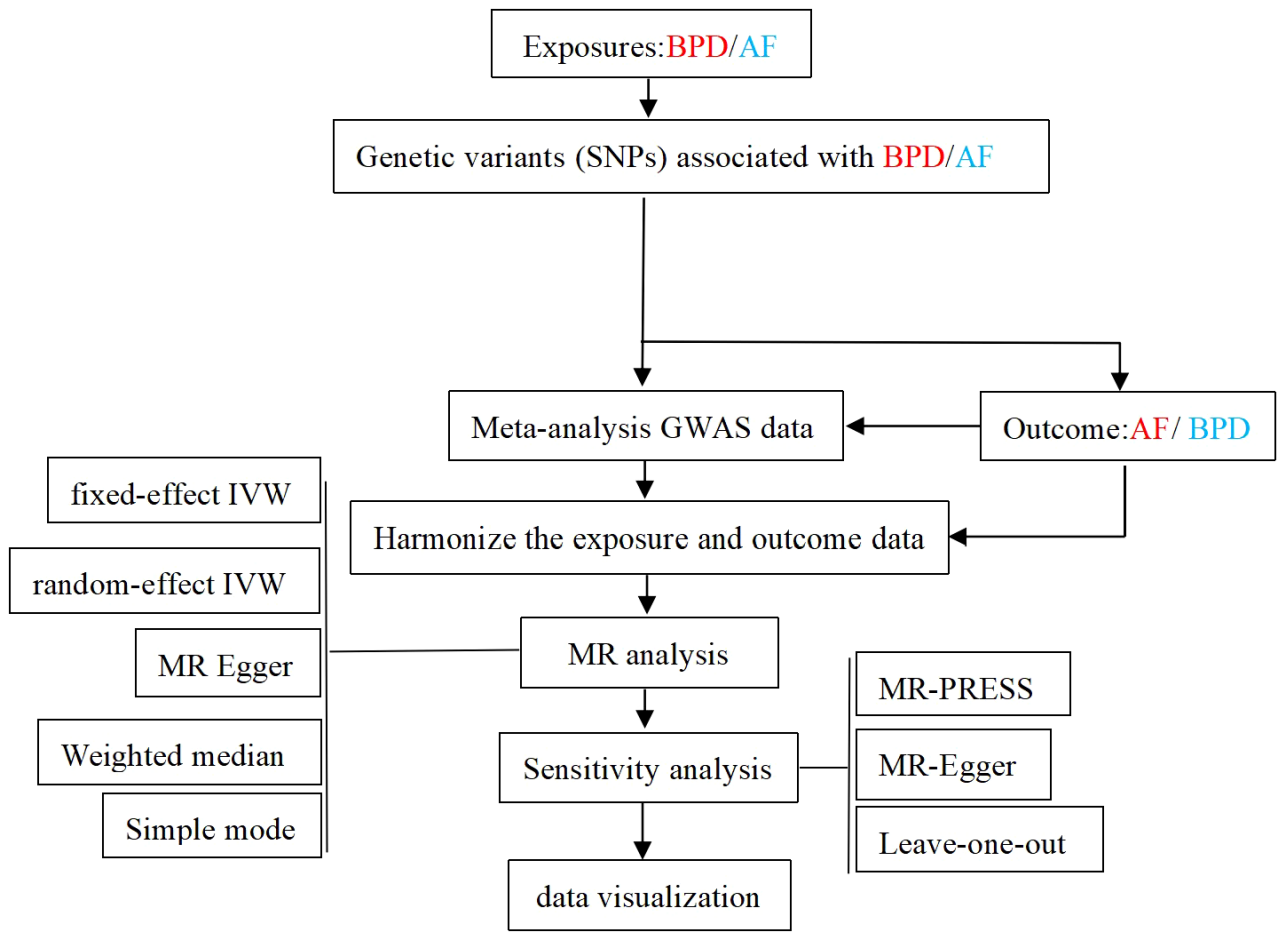
Flow chart of the overall design of the MR Analytical framework for this study. BPD, borderline personality disorder; AF, atrial fibrillation; GWAS, genome-wide association study.

Two-sample Mendelian randomization (TSMR) method was used in this study. TSMR analysis methods include fixed-effect inverse-variance weighted (IVW) method、random-effect IVW method, MR Egger regression method, Weighted median method and Simple mode method. IVW is the most important method. Heterogeneity among genetic variants was examined by heterogeneity statistics. By analyzing the p-value of the Q test of Cochrane, when *P* > 0.05, there was no significant heterogeneity. Through the MR-Egger intercept test, the horizontal multiplicity of the data can be detected, and the robustness of the results can be evaluated (27). An outlier test examines whether there are SNPS that differ significantly to further reduce this level of multiplicity. Finally, the leave-one-out method is used to determine whether the selected SNPs is reliable and stable. The results were described by odd ratio (OR) and 95% confidence interval (CI). The overall process of MR Analysis in this study is shown in **Figure 2**. In our study, R software package “TwosampleMR” was used for MR Analysis.

## 3 Result

### 3.1 The choice of SNP

SNPs for BPD and AF were derived from European studies involving both men and women. We selected 16 SNPs that met the criteria when BPD was used as exposure. We got 111 SNPs when AF was the exposure factor. Details can be found in the **Supplementary Material**.

### 3.2 Genetic liability to BPD with AF

**Figure 3:**
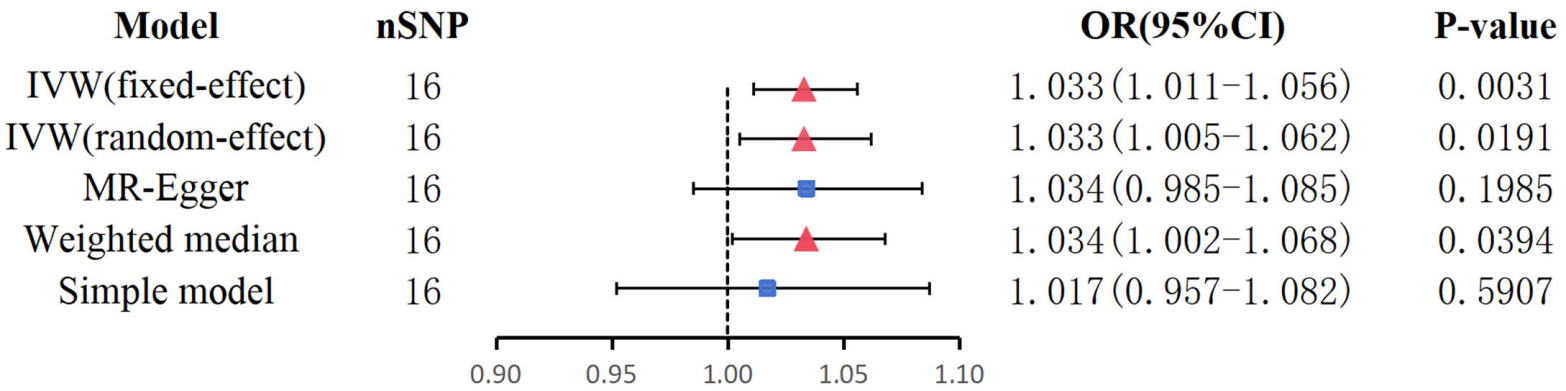
Mendelian randomization estimates of BPD on AF.

**Figure 4:**
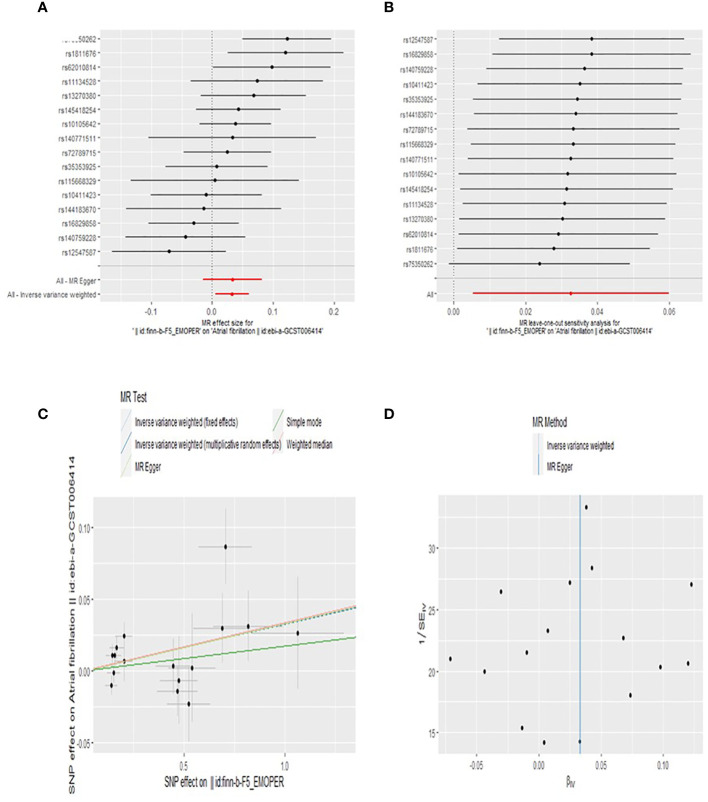
Forest plot **(A)**, sensitivity analysis **(B)**, scatter plot **(C)**, and funnel plot **(D)** of the effect of BPD on AF levels.

Our findings suggest that BPD is associated with an increased risk of AF. An observed *P* < 0.05 is considered significant evidence of causality. IVW is the main method of our research. Based on the random-effects model IVW approach, we found that an increase in BPD determined by the one standard deviation (SD) gene was causally associated with a 3.3% increase in the relative risk of AF (N = 16 SNPs; OR, 1.033; 95%CI, 1.005-1.062; *P* = 0.0191). By IVW method of fixed effect model (OR, 1.033; 95%CI, 1.011-1.056; *P* = 0.0031) and Weighted median analysis (OR, 1.034; 95%CI, 1.002-1.068; *P* = 0.0394),We also found a causal relationship between BPD and AF risk. The specific causal relationship between BPD and AF is shown in **Figure 3**. The standard forest plot shows the effect size of each SNP and its 95% confidence interval (CI) (**Figure 4A**). The leave-one-out method was used for sensitivity analysis to evaluate the reliability of the results (**Figure 4B**). Each point in the scatter plot corresponds to a SNP, showing the association between this genetic variation and BPD and AF (**Figure 4C**). Funnel plot is used to detect heterogeneity among genetic variants (**Figure 4D**).

### 3.3 Genetic liability to AF with BPD

**Figure 5:**
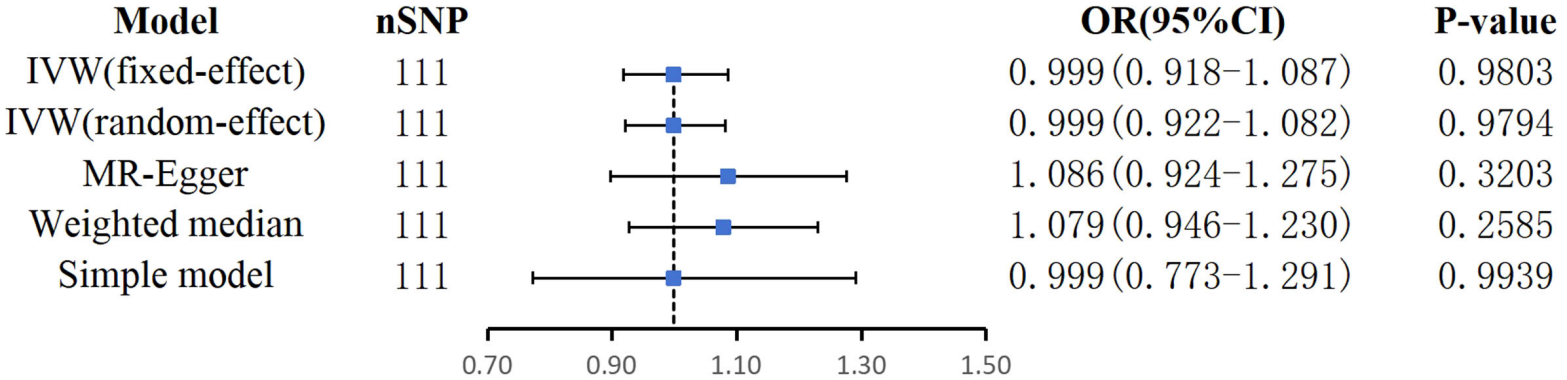
Mendelian randomization estimates of AF on BPD.

**Figure 6:**
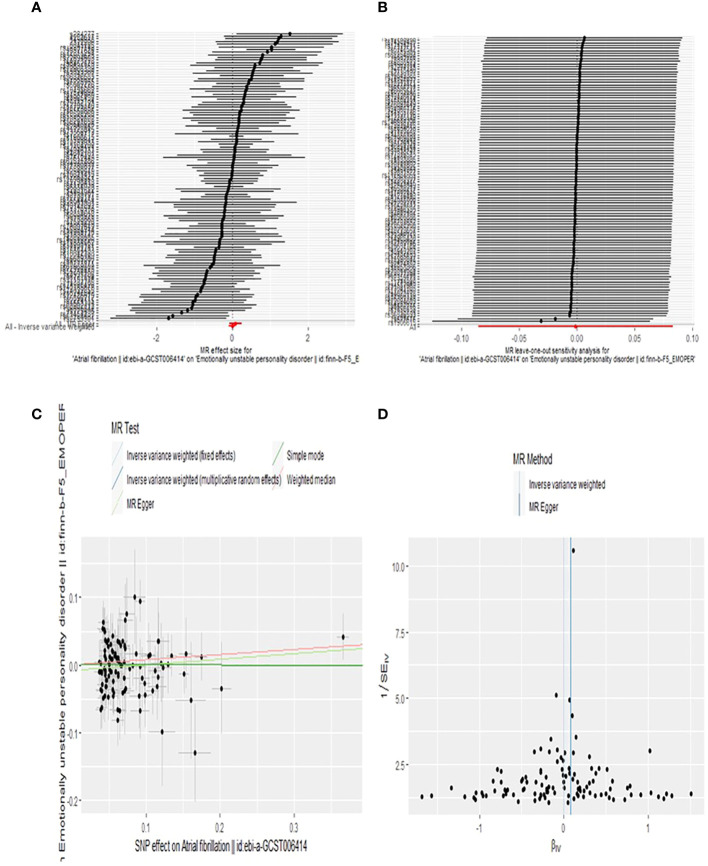
Forest plot **(A)**, sensitivity analysis **(B)**, scatter plot **(C)**, and funnel plot **(D)** of the effect of AF on BPD levels.

In reverse MR analysis, none of the five MR methods supported a causal relationship between AF genetic susceptibility and BPD (**Figure 5**). Details of MR Estimates and sensitivity analyses can be found in **Figure 6**.

### 3.4 Sensitivity analysis

We used mr_pleiotropy_test as the core analysis method to detect horizontal pleiotropy. The intercept term of the MR-Egger method was statistically tested. If there was no statistical difference, that is, *P* > 0.05, horizontal pleiotropy could not be considered. We also used the MR-PRESSO global test to detect horizontal pleiotropy. The leave-one-out method was applied to remove each SNP one by one, and then the meta effect of the remaining SNPs was calculated to observe whether the results would change significantly after the removal of a particular SNP. In the MR study of AF as an outcome, the sensitivity analysis is detailed in **Table 2**. Sensitivity analysis of AF as exposure in MR study is detailed in **Supplementary Materials**.

**Table 2:** Sensitive analyses for the Mendelian randomization analysis between BPD and AF.

Outcomes	Heterogeneity test (outliers-corrected)						Pleiotropy test (outliers-corrected)		Outiler
	MR Egger			IVW (Inverse variance weighted)			MR-PRESSO global test P-value	MR-Egger intercept test P-value	
	Cochran’s Q	Degrees of Freedom	Cochran’s Q P-value	Cochran’s Q	Degrees of Freedom	Cochran’s Q P-value			
AF	23.928	14	0.047	23.930	15	0.066	0.075	0.975	NA

BPD, borderline personality disorder; AF, atrial fibrillation; NA, not applicable.

## 4 Discussion

To our knowledge, this is the first MR analysis to assess the causal relationship between BPD and the risk of AF. We have two new findings in European populations. On the one hand, our study suggests a causal relationship between genetic susceptibility to BPD and an increased risk of AF. On the other hand, there is no evidence to support a causal relationship between AF and the risk of BPD. However, due to certain limitations of MR studies, these results must be interpreted with caution.

AF is one of the most common persistent arrhythmia. Mental disorders such as anxiety, depression and stress may activate the autonomic nervous system, which may be closely related to the development of AF. Previous studies have systematically and prospectively revealed that negative emotions such as stress, sadness, anger, and anxiety can induce AF, while happiness has a protective effect on AF (28). A systematic review noted that AF imposes significant psychosocial burdens on individuals, including depression and anxiety, as well as impaired quality of life in people with AF (29). A meta-analysis of 2017,276 participants (222,253 with anxiety disorders) from 46 cohorts showed that anxiety was not associated with AF [relative risk(RR), 1.27; 95%CI, 0.90-1.80] (30). Studies have confirmed that 20-40% of AF patients are found to have high levels of depression. Depression significantly increased the 10-year cumulative incidence of AF (from 1.92% to 4.44%) (13). A descriptive study involving 126 patients undergoing coronary artery bypass grafting (CABG) found that the mean trait anxiety scale score of patients with postoperative atrial fibrillation was 40.2 ± 7.8, with a statistically significant difference (16). A meta-analysis involving 5,329,908 participants clearly indicated that anxiety increased the risk of AF by 10% [hazard ratios(HRs) 1.10; 95%CI, 1.02-1.19; *P* = 0.013; N = 235,599 in 6 studies]. Anger increased the risk of AF by 15% (HR, 1.15; 95%CI 1.04-1.26; *P* = 0.04, N = 21,791 in 3 studies). Depression increased the risk of AF by 25% (HR, 1.25; 95%CI, 1.12-1.39; *P* < 0.001; N = 5,160,247 in 6 studies). Work stress increased the risk of AF by 18% (HR, 1.18; 95%CI,1.05-1.32; *P* = 0.004; N = 51,664 in 4 studies) (31). At the same time, many studies have shown how to influence the negative emotions of patients with AF to improve the prognosis and improve the quality of life of patients. Randomized studies in Australia have reported that improvement in psychological symptoms of anxiety and depression can be observed in patients with symptomatic AF treated with catheter ablation (32). Lakkireddy et al. have shown that yoga therapy improves depression, anxiety, blood pressure and resting heart rate, as well as quality of life in patients with paroxysmal AF (33). While several past observational studies have found a link between mental disorders and AF, the findings have been inconsistent. A large population survey suggests otherwise, that symptoms of depression and anxiety are not associated with an increased incidence of AF (34).

As mentioned above, there have been numerous studies that have linked AF to negative emotions such as depression and anxiety or psychiatric disorders, but the specific nature of their relationship is unclear. The current study also could not shed light on a key question: whether the mental disorder occurred before or after AF, or whether they affected each other at the same time. BPD, centered on emotional dysregulation, can be composed of many symptoms such as depression, anxiety, stress, and suicidal tendencies (20, 35, 36). The multiple manifestations of these unstable personality traits are similar to many mental disorders of AF. Our study specifically identified BPD as an exposure factor and concluded that BPD may predate and lead to AF.

Several studies have shown that patients with BPD exhibit fewer Respiratory sinus arrhythmia (RSA) than healthy individuals, a result that can be explained by reduced parasympathetic activity in BPD patients (37–39). We found that no experts had conducted systematic studies on the relationship between BPD and other arrhythmias. Our study was a MR study of the causal relationship between BPD and AF at the genetic level. It has been documented that inflammation is a driver of AF (40). Similarly, some experts believe that BPD may exhibit a pro-inflammatory state (41). Inflammation may be a mediating factor between BPD and AF. The TSMR analysis we performed was sufficient to validate the most direct causal relationship between BPD and AF, without further exploring how this causal relationship is affected step by step.

Finally, it should be noted that in the course of our study, we used a more relaxed threshold (*P* < 5×10^-6^) to select instrumental variables for BPD. Although this improves the statistical efficiency, it is more likely to introduce multi-effect instrumental variables. Although we performed multiple sensitivity analyses, each SNP independently affected BPD and AF, which reduced the reliability of the results.

## 5 Strengths and limitations

To our knowledge, our study is the first to use MR Analysis to explore the causal relationship between BPD and AF. TSMR analysis has several advantages (1): Compared with traditional observational studies, MR Method reduces the influence of confounding factors and reverse causality (2); We have strictly identified the SNPs selection to reduce the sampling bias; (3) The data we used are all from European populations, which reduces the influence of population stratification to a certain extent.

Our study also has some limitations: (1) Due to the lack of individual information in the samples, we could not stratify the analysis of age, and AF subtypes; (2) BPD is more common in women, and we did not stratify our study subjects by gender;(3) The samples we studied were all of European ancestry, and the conclusions cannot be generalized to all populations; (4) Not all data employed in the GWAS diagnosed BPD patients according to Diagnostic and Statistical Manual of Mental Disorders (DSM) or International Classification of Diseases (ICD) criteria, together with the lack of adjustment for comorbidities, it is difficult to be sure that the results are specific to BPD and not just common to psychiatric disorders in general.

## 6 Conclusion

Our TSMR analysis provides genetic evidence of a causal relationship between BPD and an increased risk of AF. On the contrary, no causal relationship of AF on BPD risk was observed. Our study enhances the current understanding of the role of psychiatric disorders in AF. Our study provides evidence to support early prevention of arrhythmias in patients with BPD. Further research is now needed to explore strategies for detecting and treating BPD.

## Data availability statement

The datasets presented in this study can be found in online repositories. The names of the repository/repositories and accession number(s) can be found in the article/**Supplementary Material**.

## Author contributions

WZ: Methodology, Supervision, Writing – review & editing, Validation. ZW: Conceptualization, Data curation, Methodology, Writing – original draft. HH: Formal analysis, Investigation, Project administration, Writing – review & editing. YS: Resources, Software, Writing - original draft. QW: Project administration, Supervision, Validation, Writing – original draft. MX: Supervision, Validation, Visualization, Writing – review & editing.
