# Supplementary material for: Borderline personality disorder and risk of atrial fibrillation: insights from a bidirectional Mendelian randomization study
Source: Front Psychiatry. 2024 Jul 10;15:1392605. doi: 10.3389/fpsyt.2024.1392605 (PMC11266161; doi:10.3389/fpsyt.2024.1392605)
Supplement: Supplementary file 1 [file DataSheet_1.pdf]

Supplementary Material

The MR analysis of the association of BPD with AF：Table 1-3

Table 1. Genome-wide significant SNPs for BPD

SNP	Proxy SNP	r2 for proxy	CHR	Position	EA	OA	EAF	BETA	SE	P-value	N	R2	F-statistic
rs10105642	-	-	8	91836322	A	G	0.009151	0.8186	0.1728	2.16E-06	214,816	0.012152049	2642.542561
rs10411423	-	-	19	13603320	G	A	0.7244	-0.1528	0.0332	4.29E-06	214,816	0.009322542	2021.457661
rs11134528	-	-	5	168203926	T	C	0.3566	0.1444	0.0314	4.09E-06	214,816	0.009568124	2075.22298
rs115668329	-	-	3	46109969	G	T	0.01652	0.5393	0.1167	3.79E-06	214,816	0.009450753	2049.523596
rs12547587	-	-	8	128625257	G	A	0.4736	0.1406	0.0297	2.27E-06	214,816	0.009856624	2138.418414
rs13270380	-	-	8	15105058	A	G	0.7033	-0.1565	0.0327	1.64E-06	214,816	0.010221552	2218.408045
rs140759228	-	-	4	5797334	A	G	0.02326	0.5234	0.1066	9.04E-07	214,816	0.012447614	2707.625227
rs140771511	-	-	11	133239995	C	T	0.1322	0.203	0.0438	3.64E-06	214,816	0.009455253	2050.508797
rs144183670	-	-	10	99386478	A	G	0.02578	0.4746	0.0936	3.94E-07	214,816	0.011314241	2458.270835
rs145418254	-	-	2	43784007	T	C	0.0126	0.6892	0.1414	1.09E-06	214,816	0.011819094	2569.273342
rs16829858	-	-	2	134727829	G	A	0.02472	0.4678	0.1012	3.81E-06	214,816	0.01055184	2290.855701
rs1811676	-	-	10	109923140	G	A	0.1354	0.2025	0.044	4.27E-06	214,816	0.009600944	2082.410289
rs35353925			11	132095549	A	T	0.03246	0.4475	0.0868	2.50E-07	214,816	0.012578635	2736.488185
rs62010814			15	69295864	G	A	0.2224	0.167	0.0361	3.73E-06	214,816	0.009646149	2092.310591
rs72789715	-	-	5	125929746	A	T	0.005206	1.0635	0.2286	3.27E-06	214,816	0.011715	2546.376814
rs75350262	-	-	1	10484432	A	G	0.01472	0.705	0.1319	9.00E-08	214,816	0.014417027	3142.281597

Table 2. Instrumental variables used in MR analysis of the association between BPD and AF

SNP	CHR	POS	EA	OA	EAF	SNP-Exposure (BPD)			SNP-Outcome (AF)		
						BETA	SE	p	BETA	SE	p
rs10105642	8	91836322	A	G	0.009151	0.8186	0.1728	2.16E-06	0.0313	0.0246	0.2035
rs10411423	19	13603320	G	A	0.7244	-0.1528	0.0332	4.29E-06	0.0015	0.0071	0.8317
rs11134528	5	168203926	T	C	0.3566	0.1444	0.0314	4.09E-06	0.0106	0.008	0.1867
rs115668329	3	46109969	G	T	0.01652	0.5393	0.1167	3.79E-06	0.0022	0.0379	0.9535
rs12547587	8	128625257	G	A	0.4736	0.1406	0.0297	2.27E-06	-0.01	0.0067	0.1367
rs13270380	8	15105058	A	G	0.7033	-0.1565	0.0327	1.64E-06	-0.0106	0.0069	0.1271
rs140759228	4	5797334	A	G	0.02326	0.5234	0.1066	9.04E-07	-0.0229	0.0262	0.3826
rs140771511	11	133239995	C	T	0.1322	0.203	0.0438	3.64E-06	0.0067	0.0142	0.6367
rs144183670	10	99386478	A	G	0.02578	0.4746	0.0936	3.94E-07	-0.0065	0.0308	0.832
rs145418254	2	43784007	T	C	0.0126	0.6892	0.1414	1.09E-06	0.0295	0.0243	0.2262
rs16829858	2	134727829	G	A	0.02472	0.4678	0.1012	3.81E-06	-0.0142	0.0177	0.4223
rs1811676	10	109923140	G	A	0.1354	0.2025	0.044	4.27E-06	0.0243	0.0098	0.0132902
rs35353925	11	132095549	A	T	0.03246	0.4475	0.0868	2.50E-07	0.0032	0.0192	0.8666
rs62010814	15	69295864	G	A	0.2224	0.167	0.0361	3.73E-06	0.0163	0.0082	0.0476398
rs72789715	5	125929746	A	T	0.005206	1.0635	0.2286	3.27E-06	0.0266	0.0391	0.4961
rs75350262	1	10484432	A	G	0.01472	0.705	0.1319	9.00E-08	0.0863	0.0261	0.000955608

Table 3. Mendelian randomization estimates between BPD and AF

Outcomes	N	Ivw (Random effect)		Ivw (Fixed effect)		Weighted median		Mr-Egger		simple_mode	
		OR (95%CI)	P-value	OR (95%CI)	P-value	OR (95%CI)	P-value	OR (95%CI)	P-value	OR (95%CI)	P-value
AF	16	1.033 1.005-1.062	0.0191	1.033 1.011-1.056	0.0031	1.034 1.002-1.068	0.0394	1.034 0.985-1.085	0.1985	1.017 0.957-1.082	0.5907

The MR analysis of the association of AF with BPD: Table 4-7

Table 4. Genome-wide significant SNPs for AF

SNP	Proxy SNP	r2 for proxy	CHR	Position	EA	OA	EAF	BETA	SE	P-value	N	R2	F-statistic
rs10141892	-	-	14	35184323	C	T	0.5833	-0.0452	0.0068	2.95E-11	1,030,836	0.000993167	1024.808262
rs10213171	-	-	4	148937537	G	C	0.0609	0.091	0.0134	1.32E-11	1,030,836	0.0009472	977.3321982
rs10458662	-	-	10	77936670	G	T	0.1722	0.0544	0.0088	6.93E-10	1,030,836	0.000843697	870.4456639
rs10520002	-	-	5	127819132	A	G	0.0988	0.0626	0.0113	2.85E-08	1,030,836	0.000697841	719.8610884
rs10520260	-	-	4	174447349	G	A	0.3214	-0.0457	0.0073	3.36E-10	1,030,836	0.000911008	939.954181
rs10753933	-	-	1	203026214	G	T	0.5518	-0.0609	0.0067	9.84E-20	1,030,836	0.001834502	1894.542313
rs10773657	-	-	12	123327900	A	C	0.862	-0.0575	0.0103	2.54E-08	1,030,836	0.000786597	811.4887823
rs10804493	-	-	3	111554426	A	G	0.6505	0.0558	0.007	1.63E-15	1,030,836	0.001415771	1461.49356
rs10821415	-	-	9	97713459	A	C	0.4132	0.0821	0.0067	2.92E-34	1,030,836	0.003268637	3380.471965
rs10842383	-	-	12	24771967	T	C	0.1478	-0.0988	0.0095	2.88E-25	1,030,836	0.002459007	2541.077038
rs11191116	-	-	10	103555611	T	C	0.348	-0.041	0.007	4.42E-09	1,030,836	0.000762824	786.9455793
rs11264280	-	-	1	154862952	T	C	0.333	0.1347	0.0071	3.07E-79	1,030,836	0.008060004	8376.037001
rs11598047	-	-	10	105342672	G	A	0.1621	0.1537	0.009	8.95E-66	1,030,836	0.006417309	6657.905906
rs11773845	-	-	7	116191301	A	C	0.5856	0.1054	0.0067	2.39E-55	1,030,836	0.005391778	5588.158578
rs117984853	-	-	6	149399100	T	G	0.1013	0.1228	0.012	1.34E-24	1,030,836	0.002745686	2838.139429
rs12245149	-	-	10	65321147	A	C	0.4739	-0.047	0.0067	1.66E-12	1,030,836	0.00110149	1136.70584
rs12426679	-	-	12	76237987	T	C	0.5278	-0.0391	0.0067	4.95E-09	1,030,836	0.000762042	786.1378204
rs1278493	-	-	3	135814009	A	G	0.5645	-0.0389	0.0068	8.77E-09	1,030,836	0.000744014	767.5263248
rs133885	-	-	22	26159289	A	G	0.4377	0.0405	0.0068	2.22E-09	1,030,836	0.000807392	832.960092
rs140185678	-	-	16	2003016	A	G	0.0351	0.1659	0.0218	2.43E-14	1,030,836	0.001864285	1925.35727
rs1458038	-	-	4	81164723	T	C	0.3087	0.0434	0.0072	1.74E-09	1,030,836	0.00080392	829.374453
rs146518726	-	-	1	51535039	A	G	0.0328	0.1605	0.0207	8.27E-15	1,030,836	0.001634445	1687.599337
rs1563304	-	-	17	44874453	T	C	0.178	0.0644	0.0092	2.56E-12	1,030,836	0.00121365	1252.592152
rs17171711	-	-	5	137364795	T	C	0.1775	0.1086	0.0087	1.95E-35	1,030,836	0.003443689	3562.138496
rs17380837	-	-	12	26345526	T	C	0.307	-0.0501	0.0072	4.80E-12	1,030,836	0.001068014	1102.12251
rs1838747	-	-	5	114426668	G	A	0.4954	0.0391	0.0067	4.13E-09	1,030,836	0.00076434	788.5106601
rs1906615	-	-	4	111701798	T	G	0.1991	0.3658	0.0081	1.00E-200	1,030,836	0.042674354	45951.10849
rs2031522	-	-	6	87821501	G	A	0.3764	-0.0436	0.0068	1.47E-10	1,030,836	0.000892398	920.7360902
rs2274115	-	-	9	139094773	G	A	0.7003	0.0487	0.0076	1.69E-10	1,030,836	0.00099554	1027.259331

rs2288327	-	-	2	179411665	G	A	0.1564	0.0919	0.0089	7.26E-25	1,030,836	0.002228611	2302.459641
rs2359171	-	-	16	73053022	A	T	0.176	0.1746	0.0086	4.65E-91	1,030,836	0.00884216	9196.112333
rs2540949	-	-	2	65284231	T	A	0.3847	-0.0659	0.0068	2.95E-22	1,030,836	0.002055938	2123.696553
rs2738413	-	-	14	64679960	G	A	0.5049	-0.0778	0.0067	2.55E-31	1,030,836	0.003026129	3128.905487
rs2739197	-	-	4	111543323	G	C	0.2458	0.116	0.0086	3.20E-41	1,030,836	0.00498901	5168.627544
rs2739197	-	-	4	111543323	G	C	0.2458	0.116	0.0086	3.20E-41	1,030,836	0.00498901	5168.627544
rs2759301	-	-	15	80994288	A	G	0.4542	0.039	0.0067	5.04E-09	1,030,836	0.000754119	777.9581567
rs2834618	-	-	21	36119111	G	T	0.1056	-0.0944	0.0112	3.41E-17	1,030,836	0.001683332	1738.161416
rs28387148	-	-	2	127433465	T	C	0.1051	0.0741	0.0113	6.25E-11	1,030,836	0.001032865	1065.813384
rs284277	-	-	1	10790797	A	C	0.6174	-0.0422	0.0069	1.25E-09	1,030,836	0.00084133	868.0020717
rs2860482	-	-	12	57105938	C	A	0.726	-0.054	0.0076	1.21E-12	1,030,836	0.001160125	1197.285055
rs28631169	-	-	14	23888183	T	C	0.1982	0.0522	0.0084	5.35E-10	1,030,836	0.000866045	893.5229668
rs2885697	-	-	1	41544279	T	G	0.6482	-0.0439	0.007	2.88E-10	1,030,836	0.000878949	906.8480494
rs3176326	-	-	6	36647289	A	G	0.1982	-0.0626	0.0085	1.42E-13	1,030,836	0.001245513	1285.518572
rs337705	-	-	5	113737062	G	T	0.3749	0.0564	0.0068	1.63E-16	1,030,836	0.001490916	1539.181579
rs34080181	-	-	3	66454191	A	G	0.379	-0.0446	0.0069	1.28E-10	1,030,836	0.000936333	966.1089215
rs34936990	-	-	10	105523416	A	G	0.1207	0.1294	0.0101	2.95E-37	1,030,836	0.003554208	3676.867237
rs34969716	-	-	6	18210109	A	G	0.3051	0.0702	0.0078	1.60E-19	1,030,836	0.002089627	2158.569004
rs35544454	-	-	2	213266003	T	A	0.1918	-0.0589	0.0087	1.10E-11	1,030,836	0.001075544	1109.900693
rs35569628	-	-	13	113872712	C	T	0.223	-0.0452	0.008	1.38E-08	1,030,836	0.000707999	730.3466989
rs35963991	-	-	8	11495702	T	G	0.1494	0.0525	0.0095	2.80E-08	1,030,836	0.000700527	722.6327735
rs3943207	-	-	8	21845619	T	G	0.1167	-0.0638	0.0103	6.92E-10	1,030,836	0.000839171	865.7724749
rs4073778	-	-	1	116297758	A	C	0.5639	0.0486	0.0067	4.96E-13	1,030,836	0.001161691	1198.903586
rs4252627	-	-	17	37868715	T	C	0.6679	-0.0415	0.0071	5.63E-09	1,030,836	0.000764023	788.1832122
rs4587869	-	-	14	32992334	C	G	0.2849	0.0716	0.0077	1.19E-20	1,030,836	0.002088889	2157.804744
rs4642101	-	-	3	12843862	G	T	0.6397	0.0706	0.0069	2.95E-24	1,030,836	0.00229763	2373.929083
rs464901	-	-	22	18597502	C	T	0.3353	-0.0508	0.0072	1.53E-12	1,030,836	0.001150315	1187.149051
rs4757877	-	-	11	20010291	G	A	0.7552	-0.0723	0.0078	2.93E-20	1,030,836	0.001932769	1996.222391
rs4935786	-	-	11	121661507	A	T	0.7327	-0.0463	0.0079	4.85E-09	1,030,836	0.000839686	866.3047323
rs4946333	-	-	6	118565665	G	A	0.4897	0.0639	0.0066	5.47E-22	1,030,836	0.002040739	2107.964564
rs4965430	-	-	15	99268850	G	C	0.6136	-0.0441	0.0069	1.26E-10	1,030,836	0.00092221	951.5225238
rs55734480	-	-	7	14372009	A	G	0.2494	0.0548	0.0078	2.20E-12	1,030,836	0.001124336	1160.308367
rs55985730	-	-	7	128417044	G	T	0.06	0.0867	0.0149	5.24E-09	1,030,836	0.000847905	874.7912407
rs56201652	-	-	7	92278116	A	G	0.267	-0.0531	0.0075	1.74E-12	1,030,836	0.001103657	1138.944562
rs56326533	-	-	2	201168758	C	T	0.3919	0.0685	0.0068	6.28E-24	1,030,836	0.002236461	2310.587984
rs577676	-	-	1	170587340	T	C	0.4383	-0.0923	0.0067	1.62E-43	1,030,836	0.004194781	4342.338035
rs60902112	-	-	3	194800853	T	C	0.2262	0.0445	0.0079	1.72E-08	1,030,836	0.00069322	715.0908882
rs62254082	-	-	3	69417585	C	T	0.3865	0.0404	0.007	6.34E-09	1,030,836	0.000774028	798.512671
rs62377206	-	-	5	168383543	A	G	0.0554	0.0846	0.0147	8.21E-09	1,030,836	0.00074908	772.756391
rs62521286	-	-	8	124551975	G	A	0.0663	0.1202	0.0135	4.50E-19	1,030,836	0.001788792	1847.251853
rs6462079	-	-	7	28415827	A	G	0.7208	0.0466	0.0076	8.79E-10	1,030,836	0.000874041	901.7798207
rs6546620	-	-	2	26159940	C	T	0.7999	0.0602	0.0086	3.19E-12	1,030,836	0.001160128	1197.287994
rs6560886	-	-	12	133150210	C	T	0.7884	0.051	0.009	1.49E-08	1,030,836	0.000867826	895.3615018
rs6580277	-	-	5	142818123	G	A	0.2369	0.067	0.0079	1.64E-17	1,030,836	0.001623028	1675.792702

rs6596717	-	-	5	106427609	A	C	0.6049	-0.0404	0.0068	3.00E-09	1,030,836	0.000780159	804.8427303
rs6665642	-	-	1	154802139	T	C	0.1176	-0.062	0.0112	3.06E-08	1,030,836	0.000797786	823.0411368
rs6689306	-	-	1	154395946	G	A	0.5872	-0.046	0.0068	1.36E-11	1,030,836	0.001025821	1058.536568
rs6747542	-	-	2	70106832	C	T	0.4642	-0.0554	0.0067	1.10E-16	1,030,836	0.001526713	1576.193944
rs6771054	-	-	3	89489529	C	T	0.4035	-0.0457	0.0068	2.42E-11	1,030,836	0.001005348	1037.389753
rs6790396	-	-	3	38771925	G	C	0.5959	0.0627	0.0068	2.40E-20	1,030,836	0.001893334	1955.415672
rs67969609	-	-	2	145760353	G	C	0.071	0.0711	0.0126	1.71E-08	1,030,836	0.000666873	687.8942991
rs6838973	-	-	4	111765495	T	C	0.4406	-0.1514	0.0067	1.03E-111	1,030,836	0.011299226	11780.73973
rs6882776	-	-	5	172664163	A	G	0.2835	-0.0711	0.0074	9.64E-22	1,030,836	0.002053707	2121.387572
rs6994744	-	-	8	141740868	C	A	0.4954	0.0405	0.0066	1.09E-09	1,030,836	0.000820056	846.0349742
rs71454237	-	-	12	70013415	A	G	0.209	-0.062	0.0084	1.78E-13	1,030,836	0.001270972	1311.828936
rs7172038	-	-	15	73667255	G	T	0.1597	0.112	0.0089	4.78E-36	1,030,836	0.003366707	3482.239714
rs7224711	-	-	17	76772288	T	C	0.5222	-0.0365	0.0066	3.72E-08	1,030,836	0.000664812	685.7665414
rs7225165	-	-	17	1309850	A	G	0.1133	-0.0655	0.0111	3.20E-09	1,030,836	0.000862024	889.3700126
rs72694603	-	-	1	112458893	T	C	0.3147	-0.0553	0.0072	2.26E-14	1,030,836	0.001319039	1361.506431
rs72700114	-	-	1	170193825	C	G	0.0756	0.2021	0.013	3.29E-54	1,030,836	0.005708794	5918.606831
rs72811294	-	-	17	12618680	C	G	0.1131	-0.072	0.0106	9.67E-12	1,030,836	0.001039997	1073.180772
rs72926475	-	-	2	86594487	A	G	0.1228	-0.0683	0.0102	2.37E-11	1,030,836	0.001005005	1037.035957
rs72966339	-	-	6	122398241	T	C	0.3679	-0.0616	0.0069	7.42E-19	1,030,836	0.001764847	1822.480428
rs73041705	-	-	3	24463235	C	T	0.2985	-0.0443	0.0073	1.55E-09	1,030,836	0.000821882	847.9207806
rs73366713	-	-	6	16415751	A	G	0.1396	-0.1035	0.0099	1.53E-25	1,030,836	0.002573336	2659.526217
rs74500426	-	-	4	174642789	T	G	0.0764	-0.0921	0.0127	4.29E-13	1,030,836	0.001197089	1235.479302
rs74832855	-	-	1	170171598	G	A	0.0369	0.1216	0.018	1.43E-11	1,030,836	0.001050981	1084.52684
rs74884082	-	-	14	73249419	T	C	0.2495	-0.0493	0.0078	3.48E-10	1,030,836	0.000910217	939.1377491
rs7508	-	-	8	17913970	A	G	0.7109	0.0711	0.0075	1.69E-21	1,030,836	0.002077906	2146.43578
rs7529220	-	-	1	22282619	C	T	0.8469	0.0621	0.0098	1.98E-10	1,030,836	0.001000047	1031.91467
rs7574892	-	-	2	175512820	A	G	0.4847	0.0552	0.0067	1.98E-16	1,030,836	0.001522093	1571.41751
rs76097649	-	-	11	128764570	A	G	0.0933	0.1151	0.0124	1.26E-20	1,030,836	0.002241434	2315.736664
rs7612445	-	-	3	179172979	T	G	0.1879	0.0493	0.0084	4.81E-09	1,030,836	0.000741754	765.1932294
rs77316573	-	-	16	2265271	T	C	0.1991	0.0529	0.0089	3.26E-09	1,030,836	0.000892464	920.8044157
rs775498	-	-	12	70071513	G	A	0.2798	0.0423	0.0074	1.05E-08	1,030,836	0.000721127	743.8983345
rs7789146	-	-	7	150661409	A	G	0.1787	-0.0584	0.0087	2.12E-11	1,030,836	0.001001111	1033.013017
rs7915134	-	-	10	75420180	T	C	0.1439	-0.1168	0.0095	1.42E-34	1,030,836	0.003361251	3476.577686
rs79187193	-	-	1	147255831	A	G	0.0569	-0.1162	0.0153	3.15E-14	1,030,836	0.001449146	1495.997302
rs8088085	-	-	18	48708548	C	A	0.4646	-0.0365	0.0067	4.79E-08	1,030,836	0.000662786	683.6754278
rs883079	-	-	12	114793240	T	C	0.7074	0.0981	0.0074	2.84E-40	1,030,836	0.00398389	4123.155931
rs9506925	-	-	13	23368943	T	C	0.2669	0.0449	0.0075	2.72E-09	1,030,836	0.000788923	813.890474
rs9953366	-	-	18	46474192	C	T	0.6631	0.049	0.0073	1.82E-11	1,030,836	0.001072759	1107.024092

Table 4. Instrumental variables used in MR analysis of the association between AF and BPD

SNP	CHR	POS	EA	OA	EAF	SNP-Exposure (AF)			SNP-Outcome (BPD)		
						BETA	SE	p	BETA	SE	p
rs10141892	14	35184323	C	T	0.5833	-0.0452	0.0068	2.95E-11	0.0087	0.0312	0.7796
rs10213171	4	148937537	G	C	0.0609	0.091	0.0134	1.32E-11	-0.0458	0.0485	0.345
rs10458662	10	77936670	G	T	0.1722	0.0544	0.0088	6.93E-10	0.0204	0.0415	0.623499
rs10520002	5	127819132	A	G	0.0988	0.0626	0.0113	2.85E-08	0.0107	0.0474	0.822
rs10520260	4	174447349	G	A	0.3214	-0.0457	0.0073	3.36E-10	-0.0088	0.0307	0.7733
rs10753933	1	203026214	G	T	0.5518	-0.0609	0.0067	9.84E-20	-0.0064	0.0311	0.8363
rs10773657	12	123327900	A	C	0.862	-0.0575	0.0103	2.54E-08	-0.0137	0.0387	0.7228
rs10804493	3	111554426	A	G	0.6505	0.0558	0.007	1.63E-15	-0.0245	0.0303	0.4184
rs10821415	9	97713459	A	C	0.4132	0.0821	0.0067	2.92E-34	-6.00E-04	0.0309	0.9849
rs10842383	12	24771967	T	C	0.1478	-0.0988	0.0095	2.88E-25	0.0449	0.0485	0.3547
rs11191116	10	103555611	T	C	0.348	-0.041	0.007	4.42E-09	0.0295	0.031	0.3415
rs11264280	1	154862952	T	C	0.333	0.1347	0.0071	3.07E-79	0.0134	0.0311	0.666
rs11598047	10	105342672	G	A	0.1621	0.1537	0.009	8.95E-66	0.017	0.052	0.7443
rs11773845	7	116191301	A	C	0.5856	0.1054	0.0067	2.39E-55	0.0149	0.0299	0.617
rs117984853	6	149399100	T	G	0.1013	0.1228	0.012	1.34E-24	-0.0037	0.0447	0.9349
rs12245149	10	65321147	A	C	0.4739	-0.047	0.0067	1.66E-12	0.0222	0.0297	0.4558
rs12426679	12	76237987	T	C	0.5278	-0.0391	0.0067	4.95E-09	0.036	0.0298	0.2265
rs1278493	3	135814009	A	G	0.5645	-0.0389	0.0068	8.77E-09	-0.0021	0.0299	0.9437
rs133885	22	26159289	A	G	0.4377	0.0405	0.0068	2.22E-09	-0.0095	0.0299	0.7501
rs140185678	16	2003016	A	G	0.0351	0.1659	0.0218	2.43E-14	-0.1307	0.0721	0.0696594
rs1458038	4	81164723	T	C	0.3087	0.0434	0.0072	1.74E-09	0.0529	0.0312	0.0902402
rs146518726	1	51535039	A	G	0.0328	0.1605	0.0207	8.27E-15	-0.0529	0.087	0.5429
rs1563304	17	44874453	T	C	0.178	0.0644	0.0092	2.56E-12	-0.0671	0.0447	0.1335
rs17171711	5	137364795	T	C	0.1775	0.1086	0.0087	1.95E-35	-0.0382	0.0366	0.2966
rs17380837	12	26345526	T	C	0.307	-0.0501	0.0072	4.80E-12	-3.00E-04	0.0309	0.9923
rs1838747	5	114426668	G	A	0.4954	0.0391	0.0067	4.13E-09	-0.0108	0.0299	0.719201
rs1906615	4	111701798	T	G	0.1991	0.3658	0.0081	1.00E-200	0.0418	0.0346	0.2282
rs2031522	6	87821501	G	A	0.3764	-0.0436	0.0068	1.47E-10	0.0049	0.0308	0.8726
rs2274115	9	139094773	G	A	0.7003	0.0487	0.0076	1.69E-10	-0.0474	0.0337	0.16
rs2288327	2	179411665	G	A	0.1564	0.0919	0.0089	7.26E-25	-0.0018	0.0396	0.9631
rs2359171	16	73053022	A	T	0.176	0.1746	0.0086	4.65E-91	0.0118	0.0353	0.7377
rs2540949	2	65284231	T	A	0.3847	-0.0659	0.0068	2.95E-22	4.00E-04	0.0314	0.9905
rs2738413	14	64679960	G	A	0.5049	-0.0778	0.0067	2.55E-31	0.0061	0.0296	0.8359
rs2739197	4	111543323	G	C	0.2458	0.116	0.0086	3.20E-41	-0.018	0.0334	0.59
rs2739197	4	111543323	G	C	0.2458	0.116	0.0086	3.20E-41	0.0745	0.5322	0.8887
rs2759301	15	80994288	A	G	0.4542	0.039	0.0067	5.04E-09	-0.0657	0.0304	0.0304797
rs2834618	21	36119111	G	T	0.1056	-0.0944	0.0112	3.41E-17	0.02	0.0464	0.6673
rs28387148	2	127433465	T	C	0.1051	0.0741	0.0113	6.25E-11	0.0764	0.0523	0.1445
rs284277	1	10790797	A	C	0.6174	-0.0422	0.0069	1.25E-09	-0.0637	0.0301	0.03408
rs2860482	12	57105938	C	A	0.726	-0.054	0.0076	1.21E-12	-0.0085	0.0383	0.8238

rs28631169	14	23888183	T	C	0.1982	0.0522	0.0084	5.35E-10	0.0306	0.0452	0.4985
rs2885697	1	41544279	T	G	0.6482	-0.0439	0.007	2.88E-10	-0.0219	0.0313	0.4852
rs3176326	6	36647289	A	G	0.1982	-0.0626	0.0085	1.42E-13	0.0128	0.0394	0.745901
rs337705	5	113737062	G	T	0.3749	0.0564	0.0068	1.63E-16	-0.0418	0.0306	0.1722
rs34080181	3	66454191	A	G	0.379	-0.0446	0.0069	1.28E-10	0.0154	0.0318	0.6284
rs34936990	10	105523416	A	G	0.1207	0.1294	0.0101	2.95E-37	0.0016	0.0439	0.9713
rs34969716	6	18210109	A	G	0.3051	0.0702	0.0078	1.60E-19	-0.0193	0.032	0.5455
rs35544454	2	213266003	T	A	0.1918	-0.0589	0.0087	1.10E-11	0.0104	0.0359	0.771299
rs35569628	13	113872712	C	T	0.223	-0.0452	0.008	1.38E-08	-0.0362	0.0332	0.2759
rs35963991	8	11495702	T	G	0.1494	0.0525	0.0095	2.80E-08	-0.0093	0.0501	0.8527
rs3943207	8	21845619	T	G	0.1167	-0.0638	0.0103	6.92E-10	-0.0342	0.0514	0.5052
rs4073778	1	116297758	A	C	0.5639	0.0486	0.0067	4.96E-13	0.0367	0.0298	0.2182
rs4252627	17	37868715	T	C	0.6679	-0.0415	0.0071	5.63E-09	-0.0532	0.0316	0.0916495
rs4587869	14	32992334	C	G	0.2849	0.0716	0.0077	1.19E-20	0.0236	0.0305	0.4389
rs4642101	3	12843862	G	T	0.6397	0.0706	0.0069	2.95E-24	0.0044	0.0301	0.8842
rs464901	22	18597502	C	T	0.3353	-0.0508	0.0072	1.53E-12	0.0065	0.0317	0.8369
rs4757877	11	20010291	G	A	0.7552	-0.0723	0.0078	2.93E-20	0.0353	0.037	0.3398
rs4935786	11	121661507	A	T	0.7327	-0.0463	0.0079	4.85E-09	0.0318	0.0335	0.3429
rs4946333	6	118565665	G	A	0.4897	0.0639	0.0066	5.47E-22	0.0042	0.0295	0.8862
rs4965430	15	99268850	G	C	0.6136	-0.0441	0.0069	1.26E-10	-0.0136	0.0311	0.660999
rs55734480	7	14372009	A	G	0.2494	0.0548	0.0078	2.20E-12	-0.0365	0.0352	0.3001
rs55985730	7	128417044	G	T	0.06	0.0867	0.0149	5.24E-09	0.0166	0.0541	0.7583
rs56201652	7	92278116	A	G	0.267	-0.0531	0.0075	1.74E-12	-0.009	0.034	0.7916
rs56326533	2	201168758	C	T	0.3919	0.0685	0.0068	6.28E-24	0.0352	0.0296	0.2336
rs577676	1	170587340	T	C	0.4383	-0.0923	0.0067	1.62E-43	-0.0944	0.0306	0.00205802
rs60902112	3	194800853	T	C	0.2262	0.0445	0.0079	1.72E-08	-0.0467	0.0383	0.2226
rs62254082	3	69417585	C	T	0.3865	0.0404	0.007	6.34E-09	-0.0634	0.0309	0.0404203
rs62377206	5	168383543	A	G	0.0554	0.0846	0.0147	8.21E-09	0.1004	0.0699	0.1511
rs62521286	8	124551975	G	A	0.0663	0.1202	0.0135	4.50E-19	0.0031	0.0583	0.9571
rs6462079	7	28415827	A	G	0.7208	0.0466	0.0076	8.79E-10	0.0336	0.0396	0.3961
rs6546620	2	26159940	C	T	0.7999	0.0602	0.0086	3.19E-12	0.01	0.0328	0.7597
rs6560886	12	133150210	C	T	0.7884	0.051	0.009	1.49E-08	0.012	0.0401	0.7642
rs6580277	5	142818123	G	A	0.2369	0.067	0.0079	1.64E-17	0	0.0363	0.9999
rs6596717	5	106427609	A	C	0.6049	-0.0404	0.0068	3.00E-09	0.0388	0.0307	0.2072
rs6665642	1	154802139	T	C	0.1176	-0.062	0.0112	3.06E-08	0.0661	0.05	0.1868
rs6689306	1	154395946	G	A	0.5872	-0.046	0.0068	1.36E-11	0.0223	0.0298	0.4539
rs6747542	2	70106832	C	T	0.4642	-0.0554	0.0067	1.10E-16	0.0464	0.0296	0.1168
rs6771054	3	89489529	C	T	0.4035	-0.0457	0.0068	2.42E-11	0.0321	0.0296	0.2788
rs6790396	3	38771925	G	C	0.5959	0.0627	0.0068	2.40E-20	0.0085	0.0296	0.773101
rs67969609	2	145760353	G	C	0.071	0.0711	0.0126	1.71E-08	-0.0385	0.0652	0.555
rs6838973	4	111765495	T	C	0.4406	-0.1514	0.0067	1.03E-111	0.0137	0.0296	0.6426
rs6882776	5	172664163	A	G	0.2835	-0.0711	0.0074	9.64E-22	-0.0297	0.0302	0.3249
rs6994744	8	141740868	C	A	0.4954	0.0405	0.0066	1.09E-09	-0.0111	0.0298	0.7104
rs71454237	12	70013415	A	G	0.209	-0.062	0.0084	1.78E-13	0.0826	0.0388	0.0333304

rs7172038	15	73667255	G	T	0.1597	0.112	0.0089	4.78E-36	-0.009	0.0365	0.8041
rs7224711	17	76772288	T	C	0.5222	-0.0365	0.0066	3.72E-08	-0.0043	0.0304	0.8868
rs7225165	17	1309850	A	G	0.1133	-0.0655	0.0111	3.20E-09	-0.029	0.0487	0.552
rs72694603	1	112458893	T	C	0.3147	-0.0553	0.0072	2.26E-14	-0.0434	0.0331	0.19
rs72700114	1	170193825	C	G	0.0756	0.2021	0.013	3.29E-54	-0.0343	0.0682	0.6147
rs72811294	17	12618680	C	G	0.1131	-0.072	0.0106	9.67E-12	-0.0658	0.053	0.2141
rs72926475	2	86594487	A	G	0.1228	-0.0683	0.0102	2.37E-11	0.0408	0.0502	0.4163
rs72966339	6	122398241	T	C	0.3679	-0.0616	0.0069	7.42E-19	-0.0355	0.0326	0.2757
rs73041705	3	24463235	C	T	0.2985	-0.0443	0.0073	1.55E-09	-0.0498	0.0346	0.1499
rs73366713	6	16415751	A	G	0.1396	-0.1035	0.0099	1.53E-25	-0.0129	0.0487	0.7914
rs74500426	4	174642789	T	G	0.0764	-0.0921	0.0127	4.29E-13	0.0685	0.0411	0.0954707
rs74832855	1	170171598	G	A	0.0369	0.1216	0.018	1.43E-11	-0.0995	0.0803	0.2152
rs74884082	14	73249419	T	C	0.2495	-0.0493	0.0078	3.48E-10	0.017	0.0435	0.6964
rs7508	8	17913970	A	G	0.7109	0.0711	0.0075	1.69E-21	0.027	0.0328	0.4099
rs7529220	1	22282619	C	T	0.8469	0.0621	0.0098	1.98E-10	-0.0162	0.0361	0.6534
rs7574892	2	175512820	A	G	0.4847	0.0552	0.0067	1.98E-16	0.0191	0.0297	0.5198
rs76097649	11	128764570	A	G	0.0933	0.1151	0.0124	1.26E-20	-0.0314	0.0506	0.5349
rs7612445	3	179172979	T	G	0.1879	0.0493	0.0084	4.81E-09	0.0024	0.0472	0.9594
rs77316573	16	2265271	T	C	0.1991	0.0529	0.0089	3.26E-09	-0.0108	0.0359	0.7643
rs775498	12	70071513	G	A	0.2798	0.0423	0.0074	1.05E-08	-0.0498	0.0327	0.1277
rs7789146	7	150661409	A	G	0.1787	-0.0584	0.0087	2.12E-11	-0.0158	0.035	0.6521
rs7915134	10	75420180	T	C	0.1439	-0.1168	0.0095	1.42E-34	-0.0354	0.042	0.3993
rs79187193	1	147255831	A	G	0.0569	-0.1162	0.0153	3.15E-14	-0.0349	0.0646	0.5895
rs8088085	18	48708548	C	A	0.4646	-0.0365	0.0067	4.79E-08	0.0104	0.0298	0.728201
rs883079	12	114793240	T	C	0.7074	0.0981	0.0074	2.84E-40	-0.0267	0.0317	0.3994
rs9506925	13	23368943	T	C	0.2669	0.0449	0.0075	2.72E-09	-0.01	0.0362	0.782
rs9953366	18	46474192	C	T	0.6631	0.049	0.0073	1.82E-11	0.027	0.0309	0.3829

Table 6. Mendelian randomization estimates between AF and BPD

Outcomes	N	Ivw (Random effect)		Ivw (Fixed effect)		Weighted median		Mr-Egger		simple_mode	
		OR (95%CI)	P-value	OR (95%CI)	P-value	OR (95%CI)	P-value	OR (95%CI)	P-value	OR (95%CI)	P-value
BPD	111	0.999<br>0.922-1.082	0.979	0.999<br>0.918-1.087	0.980	1.079<br>0.946-1.230	0.258	1.086<br>0.924-1.275	0.320	0.999<br>0.773-1.291	0.994

Table 7. Sensitive analyses for the Mendelian randomization analysis between AF and BPD

Outcomes	Heterogeneity test (outliers-corrected)						Pleiotropy test (outliers-corrected)		Outiler
	MR Egger			IVW(Inverse variance weighted)			MR-PRESSO global test P-value	MR-Egger intercept test P-value	
	Cochran's Q	Degrees of Freedom	Cochran's Q P-value	Cochran's Q	Degrees of Freedom	Cochran's Q P-value			
BPD	96.876	107	0.748	98.282	108	0.738	0.732	0.238	NA

BPD, borderline personality disorder; AF, atrial fibrillation
